# The Portrayal of Cesarean Section on Instagram: Mixed Methods Social Media Analysis

**DOI:** 10.2196/46531

**Published:** 2024-09-06

**Authors:** Rana Islamiah Zahroh, Marc Cheong, Alya Hazfiarini, Martha Vazquez Corona, Fitriana Murriya Ekawati, Ova Emilia, Caroline SE Homer, Ana Pilar Betrán, Meghan A Bohren

**Affiliations:** 1 Gender and Women’s Health Unit, Nossal Institute for Global Health, School of Population and Global Health University of Melbourne Carlton, Victoria Australia; 2 Faculty of Engineering and Information Technology University of Melbourne Melbourne, Victoria Australia; 3 Maternal, Child, and Adolescent Health Programme Burnet Institute Melbourne, Victoria Australia; 4 Department of Family and Community Medicine, Faculty of Medicine, Public Health and Nursing University of Gadjah Mada Yogyakarta Indonesia; 5 Department of Obstetrics and Gynaecology, Faculty of Medicine, Public Health and Nursing University of Gadjah Mada Yogyakarta Indonesia; 6 UNDP/UNFPA/UNICEF/WHO/World Bank Special Programme of Research, Development and Research Training in Human Reproduction (HRP), Department of Sexual and Reproductive Health and Research World Health Organization Geneva Switzerland

**Keywords:** cesarean section, social media analysis, maternal health, childbirth, mode of birth, instagram

## Abstract

**Background:**

Cesarean section (CS) rates in Indonesia are rapidly increasing for both sociocultural and medical reasons. However, there is limited understanding of the role that social media plays in influencing preferences regarding mode of birth (vaginal or CS). Social media provides a platform for users to seek and exchange information, including information on the mode of birth, which may help unpack social influences on health behavior.

**Objective:**

This study aims to explore how CS is portrayed on Instagram in Indonesia.

**Methods:**

We downloaded public Instagram posts from Indonesia containing CS hashtags and extracted their attributes (image, caption, hashtags, and objects and texts within images). Posts were divided into 2 periods—before COVID-19 and during COVID-19—to examine changes in CS portrayal during the pandemic. We used a mixed methods approach to analysis using text mining, descriptive statistics, and qualitative content analysis.

**Results:**

A total of 9978 posts were analyzed quantitatively, and 720 (7.22%) posts were sampled and analyzed qualitatively. The use of text (527/5913, 8.91% vs 242/4065, 5.95%; *P*<.001) and advertisement materials (411/5913, 6.95% vs 83/4065, 2.04%; *P*<.001) increased during the COVID-19 pandemic compared to before the pandemic, indicating growth of information sharing on CS over time. Posts with CS hashtags primarily promoted herbal medicine for faster recovery and services for choosing auspicious childbirth dates, encouraging elective CS. Some private health facilities offered discounts on CS for special events such as Mother’s Day and promoted techniques such as enhanced recovery after CS for comfortable, painless birth, and faster recovery after CS. Hashtags related to comfortable or painless birth (2358/5913, 39.88% vs 278/4065, 6.84%; *P*<.001), enhanced recovery after CS (124/5913, 2.1% vs 0%; *P*<.001), feng shui services (110/5913, 1.86% vs 56/4065, 1.38%; *P*=.03), names of health care providers (2974/5913, 50.3% vs 304/4065, 7.48%; *P*<.001), and names of hospitals (1460/5913, 24.69% vs 917/4065, 22.56%; *P*=.007) were more prominent during compared to before the pandemic.

**Conclusions:**

This study highlights the necessity of enforcing advertisement regulations regarding birth-related medical services in the commercial and private sectors. Enhanced health promotion efforts are crucial to ensure that women receive accurate, balanced, and appropriate information about birth options. Continuous and proactive health information dissemination from government organizations is essential to counteract biases favoring CS over vaginal birth.

## Introduction

### Background

Cesarean section (CS) rates are increasing rapidly worldwide [1,2]. Although the World Health Organization (WHO) does not recommend an ideal CS rate at the population level, in 2015, the WHO stated that CS rates of >10% are not associated with reductions in maternal and newborn mortality [3-5]. Mirroring global trends, Indonesia’s CS rates are similarly increasing, reaching >25% in 2023, with stark contrasts across the socioeconomic spectrum [6-8]. Women with higher education had a higher proportion of CS (33.4%) compared with less educated women (9.3%), demonstrating a situation of both over- and underuse of CS in Indonesia [6,7]. The reasons for the rise of CS rates are multifactorial, but nonclinical factors—which may include societal perceptions, influences, and misinformation—have been increasingly implicated [9,10]. Therefore, there is an urgent need to understand the social factors driving these rates to optimize CS use in Indonesia.

Over half of the global population uses social media, and there is evidence suggesting that exposure to information on social media may predict future health behavior [11,12]. For example, young adults exposed to tobacco use on social media are more likely engage in future smoking behavior [11]. Therefore, trends and data on social media can provide insights and potential pathways for public health advocacy and policy changes [13,14]. While pregnancy and birth information are widely available on social media and women often rely on it [15,16], there is limited understanding of how this information influences their preferences regarding birth mode. Previous studies in Brazil and Spain have found that women’s magazines portrayed CS neutrally but often inaccurately depicted associated long-term risks as lower than expected [17,18]. A study on Googling CS in Brazil revealed limited, unreliable, and incomplete information about CS [19]. Similarly, a recent social media study on Mexican media Facebook pages showed that CS was not promoted over vaginal birth and information about CS risks was generally accurate [20]. However, some comments either disregarded or defended CS risks, and others stigmatized women who chose CS [20]. While these social media studies offer insights into CS portrayal in Latin America and Europe, no studies have explored how CS is depicted on social media in Asia, including Indonesia. CS rates in Asia are expected to exceed 50% by 2030 [21], highlighting a critical need for interventions to manage these trends in resource-limited settings to prevent potential adverse outcomes.

### Objectives

Instagram is one of the most popular social media platforms, with 1 billion active users worldwide [22]. In Indonesia, Instagram holds the largest share of users and was the most popular social media platform in 2022, with >173 million active users [23,24]. Most people who use Instagram in Indonesia are women of reproductive age (between 18 and 44 years) [24]. Instagram is also the most popular social media platform among Indonesian influencers (users with large followings and well-established credibility, which allows them to influence others, aided by a considerably large audience) [25]. Analyzing images and associated text related to CS that are shared on Instagram may provide an understanding on the types of content that users are exposed to in relation to mode of birth. Therefore, this study aimed to explore how CS is portrayed on Indonesian Instagram.

## Methods

### Study Design

We adopted a mixed methods approach combining quantitative and qualitative analyses. Quantitative analysis assessed surface-level data, such as word frequency, without subjective interpretation [26]. Qualitative content analysis explored underlying meanings subjectively interpreted by the research team [27]. This approach enabled an in-depth analysis of CS portrayal at both surface and interpretative levels.

### Data Source, Data Collection, and Sampling

The data source was public Instagram posts using CS hashtags. These hashtags help users specify the topic or context of their posts and make them searchable for others [28,29]. We searched for 15 different hashtags related to CS in Bahasa Indonesia to find relevant posts (Table S1 in Multimedia Appendix 1).

We collected all public Instagram posts that used specific hashtags from August 31, 2018, to August 31, 2021. Posts were divided into 2 periods—before COVID-19 (before March 11, 2020, when the WHO declared the COVID-19 pandemic [30]) and during COVID-19 (on and after March 11, 2020)—to examine changes in CS portrayal during the pandemic. We used 4K Stogram (4K Download) to gather the data set, which is a program capable of downloading images and videos posted on Instagram users’ feeds and searching for hashtags. This software has been previously used in social media research [31-33]. We excluded posts with video content as the methods of analysis differ from those used for static images. Therefore, this study’s data set only included posted images; their caption text; and any associated metadata, including hashtags and the dates of the posts (which we refer to as “posts” throughout).

We downloaded and merged posts to remove duplicates from users who used multiple hashtags in a single post. The merged data set was then analyzed quantitatively. To increase the rigor of our quantitative analysis, we randomly selected 20 posts from each month using the R programming software (R Foundation for Statistical Computing) [34], totaling 720 posts. These sampled data were analyzed qualitatively to explore emerging themes.

### Data Analysis

#### Quantitative Analysis

For large-scale analysis of the posted images, we used Microsoft Azure Cognitive Services [35] to extract image attributes. This cloud-based artificial intelligence provides an application programming interface (API) that can be easily used without machine learning expertise. Using the Computer Vision API, we extracted objects within posts’ images (eg, buildings, people, and masks), content tags, and dominant colors to understand meanings conveyed by the posts. This is because colors may attract attention, convey specific meanings, and evoke certain emotions or motivations in the viewers [36]. We used the Optical Character Recognition API to extract text from the posts’ images. We accessed these APIs using Python (Python Software Foundation) to process and extract the attributes from the posted images.

We conducted descriptive statistical analysis to quantify the most frequent objects, content tags, colors, and texts appearing on the images and hashtags appearing on the captions. We compared the before and during COVID-19 periods using the 2-proportion *z* test (left-tailed test). Natural language processing was used to analyze the extracted text from the image using a text mining approach by examining the type of words that were frequently mentioned and clustered together to reveal topics or themes present in the posts. We visualized the word clusters using co-occurrence and correlation networks [37], which map word pairs that often appear together in the same post. The co-occurrence network included words that appeared >50 times together. In total, 50 occurrences were selected as larger (>50) or smaller (<50) showed too few or many networks, which made it difficult to see the connections between words. We examined correlation networks with a coefficient of >0.8 as a coefficient equal to 1 means that words always appear together. We conducted the analysis using the R programming platform [34] using the *widyr* package, with graphs plotted using the *ggplot2*, *igraph*, and *ggraph* packages [38].

#### Qualitative Analysis

We analyzed the randomly sampled posts using qualitative content analysis. The unit of analysis was an individual post’s image and its caption text. First, we developed the codebook, adapting it from a previous research examining the hashtag #HookahLife (public health–related issues in smoking hookahs or water pipes) on Instagram [39]. We coded the posts to the developed domains: metadata and post type, advertisements, health messages, birth stories, descriptive content of images, content of caption, tags, and positionality toward CS (the definitions can be found in Table S2 in Multimedia Appendix 1). The codebook was tested by 2 authors by independently coding 10% of the sample data set. We discussed any coding discrepancies, and the codebook was revised accordingly. Using the revised codebook, one author coded all the posts, and the coding was then reviewed by another author.

### Ethical Considerations

Ethics approval was obtained from the Office of Research Ethics and Integrity at the University of Melbourne (project ID 22782). This study used publicly available data on Instagram posted by nonprivate accounts. To ensure that private account posts were not inadvertently downloaded, we created a new Instagram account with no followers or users following it. This approach ensured that algorithmic recommendations from Instagram did not influence data collection. Social media sites, including Instagram, are prone to exhibit a “filter bubble” effect [40] that uses one’s social network and past activities to personalize the sort of content an end user sees. Privacy was ensured by anonymizing posts before data analysis and adhering to best practices, such as by ensuring no Instagram account names and images were included in this manuscript, and by paraphrasing quotes to avoid back translation and identification of the posters.

## Results

### Overview

A total of 13,451 public Instagram posts were downloaded. After removing duplicates and posts outside of the study time frame, 9978 posts remained, which were analyzed quantitatively. Of the 9978 posts, 720 (7.22%) were randomly selected to be analyzed qualitatively (Figure S1 in Multimedia Appendix 1).

### Quantitative Results

#### Most Frequent Objects, Content Tags, and Colors

The 3 most frequent objects in CS images were related to people (6576/9978, 65.91%), texts (769/9978, 7.71%), and advertisement material (ie, posters and flyers; 494/9978, 4.95%; Figure S2 in Multimedia Appendix 1). The most frequent content tags were text (7056/9978, 70.72%), suggesting that most CS posts are used for sharing information. Women (1739/9978, 17.43%) and babies (1635/9978, 16.39%) were the most common types of people in the images. White (7046/9978, 70.62%), gray (2347/9978, 23.52%), and black (1278/9978, 12.81%) were the most frequent colors used. During the pandemic, there were significant increases in the use of text (527/5913, 8.91% vs 242/4065, 5.95%; *P*<.001) and advertisement materials (411/5913, 6.95% vs 83/4065, 2.04%; *P*<.001) as image objects. Content tags revealed greater use of screenshots, infographics (eg, illustrations), and health care related images (eg, medical equipment) compared to prepandemic levels, reflecting increased information sharing on CS over time (Table 1). Brighter colors were notably more prevalent during compared to before the pandemic, such as yellow (825/5913, 13.95% vs 419/4065, 10.31%; *P*<.001), green (406/5913, 6.87% vs 152/4065, 3.74%; *P*<.001), and orange (131/5913, 2.22% vs 24/4065, 0.59%; *P*<.001; Table 1)—this trend suggests an effort to convey positivity through CS posts during the pandemic [36].

**Table 1:** Statistical comparison on object, content tag, color, bigram, and hashtag trends of use before and during the COVID-19 pandemic.

Trends		Before the COVID-19 pandemic^a^ (n=4065), n (%)	After the COVID-19 pandemic^b^ (n=5913), n (%)	*Z* score^c^	*P* value^d^
**Objects used in CS^e^ images**					
	Person	2781 (68.41)	3795 (64.18)	4.38	>.99
	Text	242 (5.95)	527 (8.91)	–5.45	<.001
	Advertisement material (ie, posters and flyers)	83 (2.04)	411 (6.95)	–11.11	<.001
	Bottle	113 (2.78)	123 (2.08)	2.26	.99
	Food	65 (1.59)	82 (1.39)	0.87	.81
	Infant bed	80 (1.97)	43 (0.73)	5.52	>.99
	Office supplies	27 (0.66)	73 (1.24)	–2.81	.003
	Chair	30 (0.74)	51 (0.86)	–0.68	.25
	Mammal	28 (0.69)	49 (0.83)	–0.79	.22
	Dining table	48 (1.18)	26 (0.44)	4.24	>.99
	Animal	30 (0.74)	41 (0.69)	0.26	.60
	Bowl	49 (1.21)	16 (0.27)	5.70	>.99
	Plant	16 (0.39)	41 (0.69)	–1.95	.02
	Bed	22 (0.54)	28 (0.47)	0.47	.68
	Emoji	8 (0.19)	33 (0.56)	–2.77	.003
	Cup	20 (0.49)	13 (0.22)	2.33	.99
	Fruit	16 (0.39)	16 (0.27)	0.86	.86
	Glasses	9 (0.22)	21 (0.36)	–1.19	.12
	Seating	9 (0.22)	17 (0.29)	–0.64	.26
	Vegetable	20 (0.49)	5 (0.08)	4.00	>.99
	Sports ball	1 (0.03)	23 (0.39)	–3.65	<.001
	Cell phone	9 (0.22)	13 (0.22)	0.02	.51
	Broccoli	18 (0.44)	4 (0.07)	3.93	>.99
	Tableware	12 (0.3)	9 (0.15)	1.53	.94
	Flower	1 (0.03)	17 (0.29)	–3.04	.001
**Content tags in CS images**					
	Text	2521 (62.02)	4535 (76.7)	–15.83	<.001
	Person	2249 (55.33)	2448 (41.4)	13.69	>.99
	Human face	1803 (44.35)	2107 (35.63)	8.77	>.99
	Screenshot	1317 (32.4)	2504 (42.35)	–10.05	<.001
	Clothing	1625 (39.98)	1967 (33.27)	6.86	>.99
	Indoor	1640 (40.34)	1383 (23.39)	18.11	>.99
	Woman	837 (20.59)	902 (15.25)	6.90	>.99
	Baby	938 (23.08)	697 (11.79)	14.97	>.99
	Font	433 (10.65)	1053 (17.81)	–9.87	<.001
	Design	313 (7.7)	1049 (17.74)	–14.35	<.001
	Toddler	685 (16.85)	416 (7.04)	15.38	>.99
	Child	720 (17.71)	356 (6.02)	18.50	>.99
	Cartoon	236 (5.81)	799 (13.51)	–12.41	<.001
	Newborn	561 (13.8)	438 (7.41)	10.45	>.99
	Wall	462 (11.37)	531 (8.98)	3.91	>.99
	Smile	462 (11.37)	522 (8.83)	4.18	>.99
	Girl	550 (13.53)	263 (4.45)	16.29	>.99
	Room	245 (6.03)	469 (7.93)	–3.67	<.001
	Illustration	146 (3.59)	566 (9.57)	–11.40	<.001
	Food	422 (10.38)	265 (4.48)	11.44	>.99
	Boy	403 (9.91)	283 (4.79)	9.95	>.99
	Medical equipment	240 (5.9)	410 (6.93)	–2.05	.02
	Health care	207 (5.09)	378 (6.39)	–2.72	.003
	Medical	197 (4.85)	410 (6.93)	–4.29	<.001
	Advertisement material (ie, posters and flyers)	124 (3.05)	386 (6.53)	–7.75	<.001
**Dominant color used in CS images**					
	White	3008 (74)	4038 (68.29)	6.15	>.99
	Gray	1218 (29.96)	1129 (19.09)	12.58	>.99
	Yellow	419 (10.31)	825 (13.95)	–5.42	<.001
	Black	645 (15.87)	633 (10.71)	7.58	>.99
	Pink	330 (8.12)	459 (7.76)	0.65	.74
	Green	152 (3.74)	406 (6.87)	–6.68	<.001
	Brown	418 (10.28)	279 (4.72)	10.71	>.99
	Blue	178 (4.38)	175 (2.96)	3.77	>.99
	Red	110 (2.71)	166 (2.81)	–0.30	.38
	Orange	24 (0.59)	131 (2.22)	–6.45	<.001
	Teal	83 (2.04)	122 (2.06)	–0.07	.47
	Purple	6 (0.15)	16 (0.27)	–1.29	.09
**Notable bigram topics in CS images**					
	Products or words related to faster CS recovery	2895 (71.22)	1266 (21.41)	49.58	>.99
	Names of health care providers	0 (0)	524 (8.86)	–19.49	<.001
	Names of hospitals	770 (18.94)	652 (11.03)	11.11	>.99
	Mental health	0 (0)	302 (5.11)	–14.63	<.001
	Breastfeeding	53 (1.3)	53 (0.9)	1.95	.97
	Postpartum weight loss	56 (1.38)	93 (1.57)	–0.79	.22
**Notable hashtag topics in CS posts**					
	Ultrasound	106 (2.61)	2399 (40.57)	–42.97	<.001
	Feng shui services	56 (1.38)	110 (1.86)	–1.85	.03
	Comfortable or painless birth	278 (6.84)	2358 (39.88)	–36.78	<.001
	ERACS^f^ technique	0 (0)	124 (2.1)	–9.29	<.001
	Contractions or labor pain	242 (5.95)	16 (0.27)	17.57	>.99
	Cheap labor	22 (0.54)	27 (0.46)	0.59	.72
	VBAC^g^	87 (2.14)	307 (5.19)	–7.69	<.001
	Advertised products	2748 (67.6)	2746 (46.44)	20.88	>.99
	CS wound	3002 (73.85)	1199 (20.28)	53.26	>.99
	Medicine for CS	2363 (58.13)	560 (9.47)	52.48	>.99
	Natural medicine for CS	1133 (27.87)	145 (2.45)	37.33	>.99
	Speedy recovery from CS	1296 (31.88)	89 (1.51)	43.12	>.99
	Names of health care providers	304 (7.48)	2974 (50.3)	–44.74	<.001
	Names of hospitals	917 (22.56)	1460 (24.69)	–2.46	.007

^a^Proportion of use before the COVID-19 pandemic.

^b^Proportion of use during the COVID-19 pandemic.

^c^We used a left-tailed test for this analysis, which means that a null hypothesis was that use before the COVID-19 pandemic was greater or equal to use during the COVID-19 pandemic (H_0_: proportion before the COVID-19 pandemic–proportion after the COVID-19 pandemic ≥0), whereas our alternative hypothesis was that use before the COVID-19 pandemic was lower than use during the COVID-19 pandemic (H_a_: proportion before the COVID-19 pandemic–proportion after the COVID-19 pandemic <0). We set the significance level at α=.05, and the critical value for a left-tailed test is *Z*=−1.64. Therefore, the rejection region for this left-tailed test was *R*=(Z:Z <–1.64).

^d^*P*<.05 means that trends of use are greater during the COVID-19 pandemic compared to before (proportion before the COVID-19 pandemic<proportion after the COVID-19 pandemic), whereas *P*≥.05 means that there is no evidence to reject the null hypothesis, which means that the proportion before the COVID-19 pandemic>proportion after the COVID-19 pandemic or there was no difference between before and during the COVID-19 pandemic other than due to chance.

^e^CS: cesarean section.

^f^ERACS: enhanced recovery after cesarean surgery.

^g^VBAC: vaginal birth after cesarean section.

#### Word Clusters in CS Images

**Figure 1:**
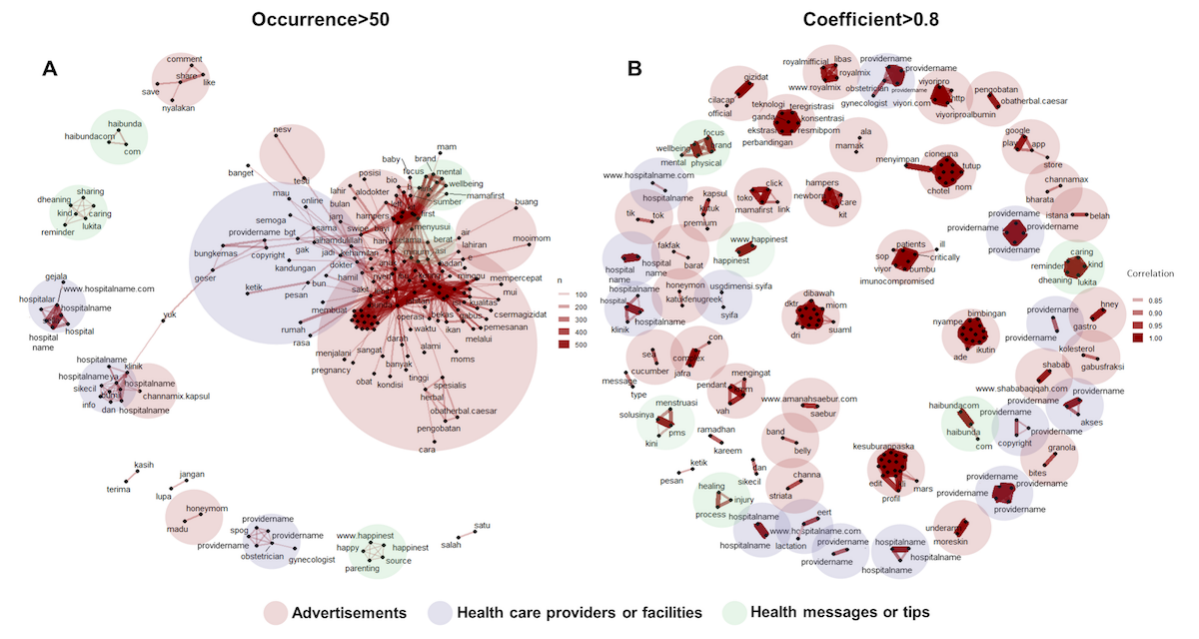
Network of word co-occurrence and correlation on cesarean section images. Panel A is a co-occurrence network presenting word pairs that occur most often (only word pairs that occur >50 times are shown); panel B shows words that occur more often together than with other words (we only show word pairs with a coefficient of >0.8; coefficient=1 means that words always appear together).

Analyzing the co-occurrence of words showed the word pairs that occurred most often in the text (Figure 1A). The co-occurrence network showed 3 main word clusters representing advertisements, health facilities and providers, and health messages. The advertisements cluster seemed to be the dominant cluster (red shade, Figure 1A). We examined the correlation of these words further by looking at words that occurred more often together than with other words (Figure 1B). From this network, we can see clearer groups of words that suggest that Instagram posts using CS words were mostly related to advertisements (red shade), followed by health facilities and providers (purple shade) and health messages (green shade). A glossary containing a sample of words in Bahasa with English translations can be found in Table S3 in Multimedia Appendix 1.

#### Most Frequent Text Appearing in CS Images

**Figure 2:**
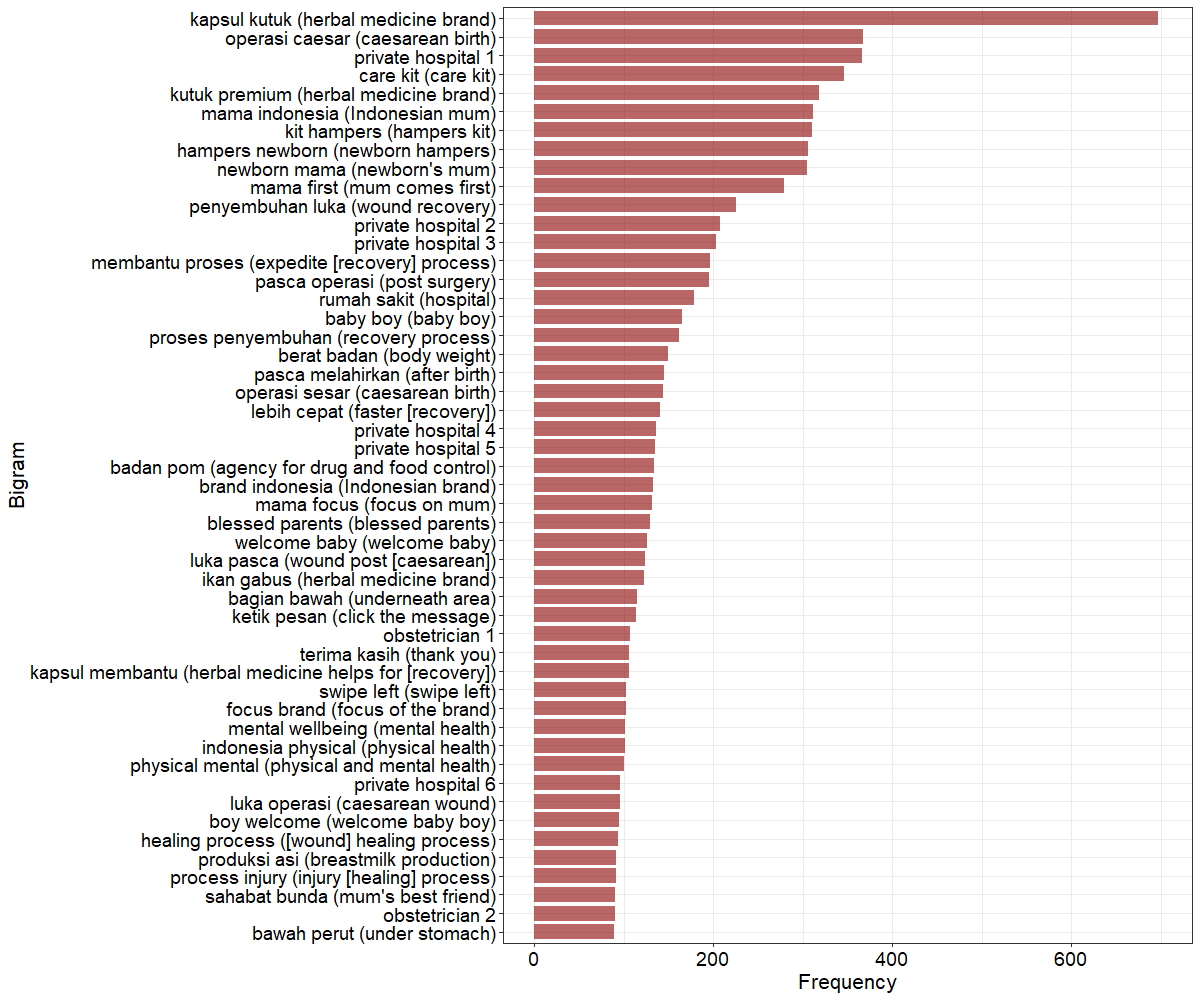
The 50 most used bigrams on cesarean section images (N=9978 posts before and during the COVID-19 pandemic). A bigram is a text comprising 2 words.

The text mining analysis explored the most used bigrams (pairs of words) appearing in CS images to see topics mentioned on the images and showed similar results to those of network analysis (Figure 2). The most frequent bigrams that appeared in CS images were mostly related to advertisements of products to accelerate CS recovery (4161/9978, 41.7%; Figure 2). Private hospital and obstetrician names (anonymized in our visualization in the interest of privacy) also appeared on this list (1946/9978, 19.5%), as well as words referring to mothers or parents (1245/9978, 12.48%), care kits for women and newborns (962/9978, 9.64%), different words for CS (511/9978, 5.12%), babies (386/9978, 3.87%), mental and physical well-being (302/9978, 3.03%), postpartum weight loss (149/9978, 1.49%), breastfeeding (91/9978, 0.91%), and others (322/9978, 3.23%) that consisted of typical Instagram words (ie, “type message,” “swipe left,” and “thank you”). The trends of observed bigrams before and during the pandemic were similar except for bigrams related to health care providers’ names (524/5913, 8.86% vs 0%; *P*<.001) and mental well-being (302/5913, 5.11% vs 0%; *P*<.001), which were more common during compared to before the COVID-19 pandemic (Table 1 and Figure S3 in Multimedia Appendix 1).

#### Most Frequent Hashtags Appearing in CS Images

**Figure 3:**
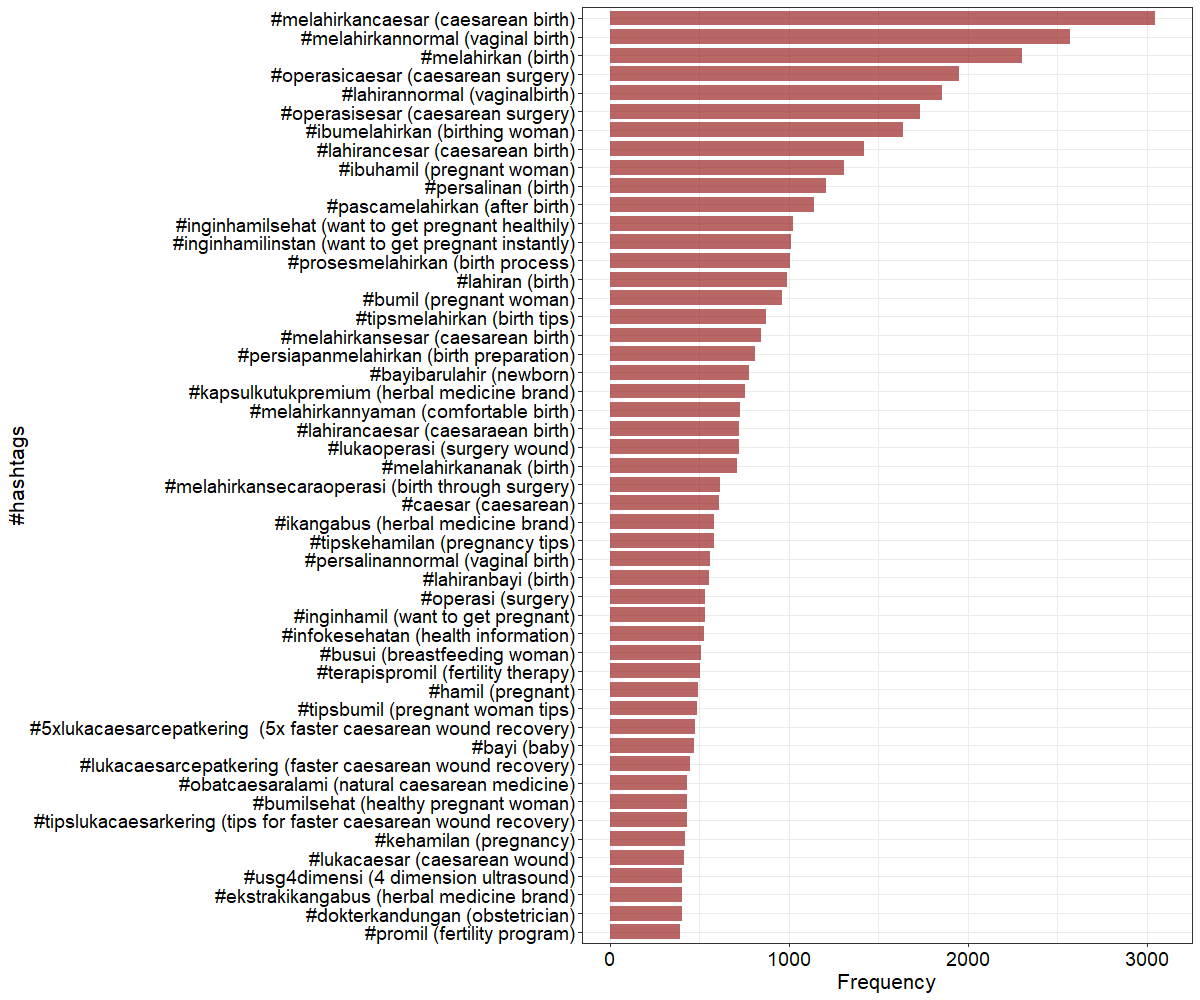
The 50 most used hashtags on cesarean section posts (N=9978 posts before and during the COVID-19 pandemic).

Multiple hashtags were commonly used in a single post. Most hashtags used on CS posts were related to mode of birth (16,473 hashtags, comprising 11,486/16,473, 69.73% instances of #CaesareanSection and 4987/16,473, 30.27% instances of #VaginalBirth), followed by birth (10,829 hashtags, comprising 7408/10,829, 68.41% instances of #Birth; 2694/10,829, 24.88% instances of #BirthTips; and 727/10,829, 6.71% instances of #ComfortableBirth; Figure 3). Other hashtags were related to pregnancy (5093 hashtags), CS recovery (4670 hashtags, including 1744/4670, 37.34% instances of #AdvertisedProduct; 1355/4670, 29.01% instances of #FasterCSRecovery; 1137/4670, 24.35% instances of #CaesareanWound; and 434/4670, 9.29% instances of #NaturalMedicine), fertility (3469 hashtags), the postpartum period (2900 hashtags), health information (529 hashtags), and obstetricians (403 hashtags). There were 2 kinds of hashtags worth highlighting: comfortable birth and faster recovery from CS, which conveyed the message that painless birth through CS and fast wound recovery from CS were possible. Comparing the top 50 hashtags before and during the COVID-19 pandemic, we found more posts about faster CS recovery before the COVID-19 pandemic and more posts about comfortable and smooth birth during the COVID-19 pandemic (Figure S4 in Multimedia Appendix 1).

**Figure 4:**
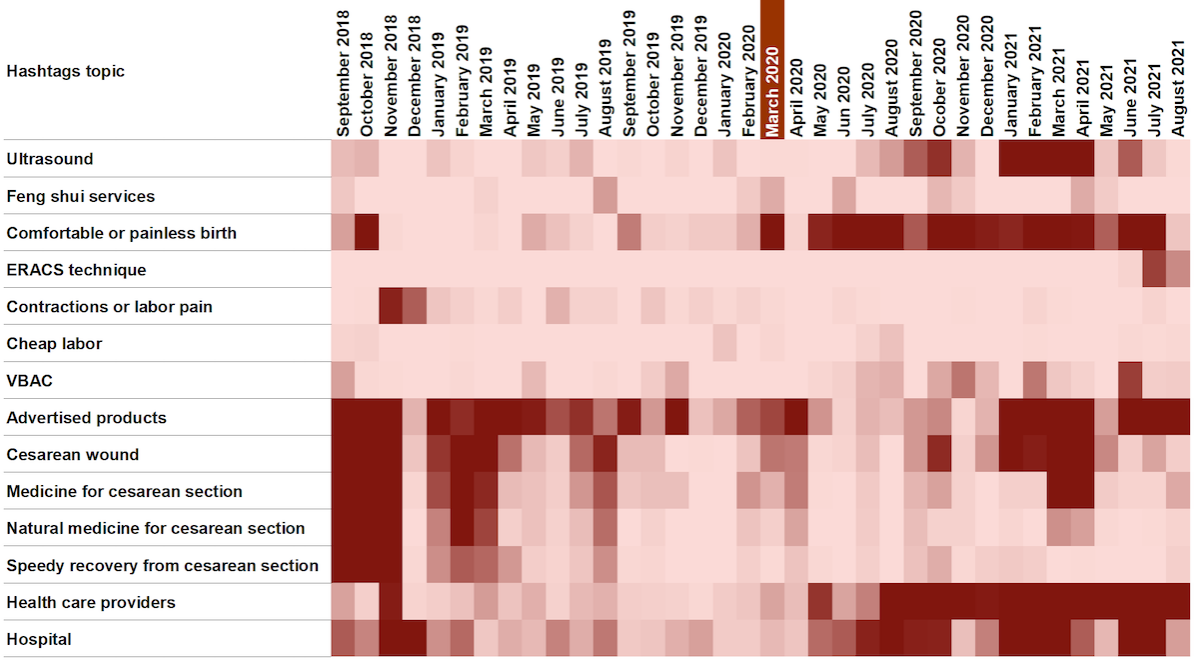
Trends in notable hashtags over time (month to month) during the study period dates. March 11, 2020, was the day the World Health Organization declared the COVID-19 pandemic [30]. ERACS: enhanced recovery after cesarean surgery; VBAC: vaginal birth after cesarean section.

As the “comfortable birth” tag seemed to appear more during the COVID-19 pandemic, we were motivated to explore the trends of notable hashtags used across time (Figure 4). The heat map in Figure 4 (darker colors denoting higher frequency) confirms that hashtags related to comfortable birth, including painless birth, as well as a clinical technique called enhanced recovery after cesarean surgery (ERACS; perioperative care initiative that may accelerate patient recovery after CS [41]), ultrasound, and names of health care providers and hospitals seem to be more prominent after the COVID-19 pandemic started. The 2-proportion *z* test analysis (Table 1) also confirmed further that there were statistically significant increases (*P*<.001) in hashtags related to comfortable or painless birth (2358/5913, 39.88% vs 278/4065, 6.84%), the ERACS technique (124/5913, 2.1% vs 0%), feng shui services (110/5913, 1.86% vs 56/4065, 1.38%), ultrasound (2399/5913, 40.57% vs 106/4065, 2.61%), vaginal birth after CS (VBAC; 307/5913, 5.19% vs 87/4065, 2.14%), names of hospitals (1460/5913, 24.69% vs 917/4065, 22.56%), and names of health care providers (2974/5913, 50.3% vs 304/4065, 7.48%) between the 2 periods. On the other hand, it was observed that hashtags related to CS recovery were greater before the pandemic, or there was no difference across the 2 periods other than due to chance (89/5913, 1.51% vs 1296/4065, 31.88%; *P*>.99).

### Qualitative Results

#### Overview

Of the 720 posts randomly sampled for qualitative analysis, advertisements were the most common posts (n=342, 47.5%), followed by health messages (n=241, 33.5%) and birth stories (n=137, 19%). There were 54.9% (395/720) of posts that used CS hashtags yet did not cover topics in relation to CS. Table S4 in Multimedia Appendix 1 presents the themes emerging from these posts.

Of the remaining 45.1% (325/720) of posts that mentioned CS explicitly, 58.2% (189/325) were posted before the COVID-19 pandemic, and 41.8% (136/325) of the posts were posted during the pandemic. Only 23.4% (76/325) of the posts referred to the location of the poster, most of whom were from Jakarta Metropolitan Area (46/76, 61%), followed by East Java (8/76, 11%), West Sumatra (8/76, 11%), South Sumatra (5/76, 7%), Central Java (5/76, 7%), Kalimantan (2/76, 3%), South Sulawesi (1/76, 1%), and Papua (1/76, 1%).

#### Advertisements

Advertisement posts aimed to advertise products or services and were made by commercial companies, individual sellers, or private health facilities. Advertisement posts were often accompanied by health messages from nontrusted sources and endorsement by influencers through their experiences in using the products or services.

#### Advertisements by Commercial Companies or Individual Sellers

CS posts advertised consumable products, wearable products, and services. Most consumable products advertised were herbal medicine, nutritional supplements, treatment oils, and drinks, whereas the wearable products were “health” belts, pendants, and corsets—all products allegedly to improve recovery after CS. These products were also advertised to balance women’s hormones, promote regular menstruation, alleviate pain, cleanse uterine blood, and restore the uterus after CS. Themes from the images, captions, and hashtags when promoting consumable and wearable products aimed to persuade women that the products were natural, safe, trusted, effective, and internationally recognized to heal the CS wound faster—emphasizing that quick CS recovery is possible. Services offered included feng shui (Chinese geomancy) consultations to choose an auspicious day of birth and free pregnancy and health consultations with non–health care providers, such as sellers of a renowned honey brand. Themes more commonly identified from advertising services were related to the importance of birth timing, suggesting that auspicious days and golden hours of birth impacted the baby’s harmony of life, future luck, and purported intelligence—encouraging planned CS to achieve this. There was no change in the types of products and advertising methods from before to during the pandemic except for postpartum fitness advertisements, which only appeared during the pandemic. These advertisements focused on postpartum body norms, encouraging women to regain their prepregnancy body shape and lose “pregnancy fat.” Posters mentioned that the adage that said that a woman with CS cannot undo her belly size is a myth and excuse and used hashtags such as #PostPartumBody, #FatBurn, #FitMom, #langsing (slim), and #sexy.

Overall, the emotions portrayed on the advertisement posts were positive, happy, and persuasive, except when risks were mentioned, in which case the emotions could be serious and tense. When analyzing the positionality regarding CS in advertisements, we found that most posts (73/101, 72.3%) implicitly encouraged CS through emphasis on the possibility of a fast recovery after CS. Only 1% (1/101) of the posts explicitly promoted CS through a feng shui promotion. Meanwhile, 16.8% (17/101) of the posts remained neutral on the matter. Notably, 2% (2/101) of the posts explicitly discouraged CS, and 9.9% (10/101) of the posts implicitly discouraged CS by highlighting associated risks.

#### Advertisements by Private Health Facilities

Posts made by private facilities typically aimed to promote their services. Some posts promoted CS uptake by giving discounts on CS costs during special events, such as during Mother’s Day, Indonesian Independence Day, and the hospital’s anniversary celebrations. These advertisements relayed the message to women that “painless and comfortable labour” (referring to CS) and “cheap and affordable CS” could be obtained at their facilities. The other posts aimed to promote their facilities by congratulating women in giving birth (all through CS) at their facility, offering free WhatsApp consultations, sharing women’s testimonials, and providing free seminars. Overall, similar advertisement posts by private hospitals or clinics were observed before and during the COVID-19 pandemic. However, during the pandemic, some posts shared women’s CS experiences with images of women or health care providers with a baby in the surgery room after CS. The hashtags used on these posts included CS-related hashtags and #SafeLabour, #ComfortableLabour, and #AlwaysSafe—relaying an overall message that CS was safe despite the COVID-19 pandemic.

The emotions portrayed on advertisement posts made by private health facilities were overall positive and happy across both periods. The positionality regarding CS on these posts was neutral (9/16, 56%) by promoting their facility instead of CS specifically, with some (5/16, 31%) explicitly encouraging it through discounted CS costs and 12% (2/16) implicitly encouraging it through the portrayal of safe CS during the pandemic.

#### Health Messages

The health message posts aimed to share information to women and the larger public. Approximately half (45/86, 52%) of the health messages were posted by health care providers or health facilities—which we refer to as “health messages from trusted sources”—whereas 48% (41/86) of the health messages were posted by non–health care providers or facilities, and these are referred to as “health messages from nontrusted sources.” The nontrusted category of messages were often posted by either personal or community groups (such as parenting, birth, or breastfeeding) accounts. Some had commercial purposes, such as promoting their Instagram accounts or certain products, yet were not as explicit compared to posts in the advertisement category.

The themes covered on health messages from both trusted and nontrusted sources were typically similar before and during the COVID-19 pandemic. Posts typically covered 3 main themes. The first theme was general statistics about childbirth and the postpartum period (shared sparingly)—which included indications, benefits, risks, the process of vaginal birth and CS, VBAC, the danger of traditional medicines and services, and mode of birth selection. The second theme was childbirth tips for women, including information on ways to induce labor, how to turn the baby’s position, preparation before CS, postpartum infection prevention, and how to support CS recovery. The third theme was motivational messages and encouragement for women to confront social stigma related to CS. These messages aimed to reassure women not to feel disheartened if they could not undergo vaginal birth, emphasizing the validity of all birth modes and promoting kindness toward other women. During the pandemic, there were reminders to undergo COVID-19 testing in the third trimester to ensure safe care for women, adherence to the COVID-19 protocol, guidelines on appropriate mask use, childbirth costs during the pandemic, and details about national health insurance coverage for CS. Interestingly, aligned with the quantitative analysis results (Figure 4), more posts were found on the promotion of CS during the pandemic in the context of the ERACS technique, which facilities and providers advertised as an “advanced technique of CS” and “painless and quick recovery labour.” Providers claimed that, with ERACS, women were able to return to their normal activity within 24 hours.

The overall emotions of these posts were positive, informative, and motivational to women. The positionality regarding CS of these posts was neutral (35/86, 41%), with some (30/86, 35%) implicitly encouraging it (when addressing the stigma associated with CS), 3% (3/86) explicitly encouraging it (mostly on promoting ERACS and risks of undergoing vaginal birth), and some (18/86, 21%) explicitly and implicitly discouraging CS when talking about CS indication and risks.

#### Birth Stories

Birth stories were mostly from women telling their birth journey, with a few posted by their partners. Approximately 20.8% (25/120) of the posts mentioned women giving birth at private health facilities, whereas 6.7% (8/120) of the posts mentioned women giving birth at public health facilities. Mode of birth was mentioned in almost all posts (116/120, 96.7%), where 90.8% (109/120) of the posts captured women with CS and only 5.8% (7/120) of the posts mentioned VBAC.

Textbox 1 shows the 5 themes identified related to CS from the birth stories before and during the pandemic, which are CS as an effort, VBAC as pride, standing up against stigma, relationships with health care providers, and powerlessness over birth. Women shared diverse perspectives on childbirth experiences, expressing pride in their efforts giving birth to their babies through CS or VBAC. Despite some women choosing CS, others desired vaginal birth and felt emotional distress when advised otherwise by health care providers. However, after birth, gratitude for a safe delivery and the baby’s safety was expressed by women regardless of mode of birth. Some women also confronted stigma, defending all birth modes and calling for kindness toward mothers who gave birth via CS. Desire for body image recovery after CS was evident with the #fashionable and #BackToSlimBody tags, showing efforts through exercise. Women also shared their relationship with their health care providers, where trust in health care providers was varied. Some women expressed gratitude for support during CS, yet dissatisfaction arose when VBAC desires clashed with providers’ pro-CS advice. Miscommunication and blame from providers were reported, prompting some women to seek more supportive and women-centered care for future births. Reflecting on their journeys, women sought empowerment and readiness for childbirth decisions, regretting past experiences and aiming for informed choices, whether CS or VBAC, to regain control over their birthing experiences.

Most posts (78/120, 65%) depicted CS as a positive birth experience, whereas the rest portrayed struggles, frustration, hopelessness, anxiety, pain, and sadness that women felt on their birth journey. The positionality regarding CS was mostly neutral (65/120, 54.2%), followed by explicitly or implicitly encouraging CS when women had negative labor experiences (38/120, 31.7%) and, finally, explicitly or implicitly discouraging CS (17/120, 14.2%) when they did not desire CS yet had CS or were discussing CS pain.

**Themes emerging from the birth stories.**

**Cesarean section (CS) as an effort**

Overall, through their birth stories women expressed their pride regarding their effort in undergoing CS for their babies by using the #perjuangan (literally, fight or effort) hashtag. While some women elected to have CS during pregnancy, some women desired vaginal birth and were heartbroken, sad, nervous, and anxious when their health care providers indicated that they should undergo CS. However, after birth, women felt gratitude to have had a safe birth—women expressed that their priority was their baby’s safety.

**Vaginal birth after CS (VBAC)** **as pride**

Women and their partners showed pride in being able to undergo VBAC. Most posts used #VBACsuccess to illustrate this. Women and their partners mentioned that they had greatly planned for women to undergo VBAC by looking for health care providers who were pro-VBAC, attending VBAC classes, praying to God, and changing their lifestyle. One woman wrote the following: “Thank God for allowing me to have VBAC with a birth gap of less than 2 years. I believe effort will not betray results or results will not betray effort.” While one woman described her experiences as the most horrible, painful, traumatic, and terrifying night of her life, others were more positive about their VBAC journey.

**Standing up against stigma**

Some posts portrayed stigma toward women who had CS. First, women and their partners felt upset with communities that devalued women who underwent CS saying that they were not “complete” or “strong” women, were “too posh to push,” and would not love their babies like women who underwent vaginal birth. Many posts advocated that all modes of birth were the same and that having a CS also required women’s sacrifice and pain and reminded other women to be kind to women who underwent CS. One woman wrote the following on her post: “I am waiting for you in a different way, I meet you through incision instead of pushing you out, I am waiting for you just like other mothers, I am a mom although I undergo CS.” Women desired acknowledgment of their experience instead of being undermined just because they underwent CS. Some posts also mentioned expectations regarding body shape after CS. Women reported desiring to lose weight and return to their “normal” body using the #fashionable and #BackToSlimBody hashtags. They depicted their efforts in returning to their body before birth by posting their pictures while exercising. One woman said the following: “Anxious and sad seeing old photos, I want to be skinny again.”

**Relationships with health care providers**

Many women who underwent CS expressed trust in health care providers’ recommendation on mode of birth and gratitude to health care providers for helping during labor. However, women who desired VBAC said that they often met health care providers who were pro-CS; thus, they said that physicians were highly against VBAC, telling the women scary stories of VBAC consequences, and made them feel rejected. Women who perceived experiences of CS or VBAC as not pleasant were often upset with their patient-physician relationship. They reported that their physicians blamed them for not being able to bear pain and push the baby out and mentioned that they would look for a physician that was pro-CS in the future.

**Powerlessness over birth**

Some women said that they were not knowledgeable about birth before, felt powerless over birth, and ended up on the surgery table: “I used to be an uneducated person, helpless about giving birth, and end up on surgery table.” Women reported regretting having CS and wishing to undergo VBAC in the future. They felt that it was important to empower themselves and ensure that their body and mind were ready for the labor process, including accepting any mode of birth they would end up choosing.

## Discussion

### Principal Findings

Our study explored how CS was portrayed on Instagram in Indonesia. We downloaded 13,451 public Instagram posts and analyzed 9978 (74.18%) posts quantitatively and 720 (5.35%) qualitatively. Quantitative analysis revealed that CS posts mostly used people, text, and advertisement materials on their images. The use of text and advertisement materials increased during the pandemic, suggesting that information sharing on CS is increasing over time. We also found that the use of infographics, brighter colors, and hashtags related to comfortable or painless birth, ERACS, VBAC, feng shui services, ultrasound, hospitals, and health care providers was more prominent during the pandemic. Qualitative analysis identified that CS was portrayed on Instagram through advertisements, health messages, and birth stories. Posts were trying to advertise products to women for faster CS recovery and services to offer women consultations in choosing an auspicious day for childbirth. Some private health facilities and health care providers explicitly promoted CS by giving discounts for CS and promoting ERACS for a comfortable, painless birth and faster recovery. Women felt powerless during their birth, unheard, and unsupported by their providers. There were also many posts that aimed to debunk stigma attributed to women who underwent CS.

Advertisement posts were the most common type of posts across time. We identified two types of sources of advertisements on Instagram: (1) those posted by health care facilities and providers and (2) those posted by companies or individual sellers. The ethics code of physicians in Indonesia [42] and Ministry of Health regulations on advertisement and publication of health services [43] do not allow health care providers in Indonesia to intentionally advertise their services or any products or techniques for profit making, including promoting discounts on health service delivery [42,43]. However, this study has shown that private health facilities and health care providers actively promoted their services on Instagram, including CS. For example, cheap CS was promoted as being accessible to women on Mother’s Day and Indonesian Independence Day. The regulations from the Ministry of Health provide detailed consequences that providers may be subject to when they are involved in advertisement, from administrative action to revocation of practice license [43]. However, given how these advertisements are actively promoted on Instagram, policies and monitoring efforts regulating advertisements for birth by health care providers and facilities and their enforcement maybe a cornerstone to ensure that women are exposed to credible information.

Our study also revealed that health care providers and facilities actively promoted ERACS uptake. ERACS is derived from the enhanced recovery after surgery technique, and the term was first used for patients undergoing colorectal surgery as a series of evidence-based interventions used for perioperative care to accelerate patient recovery after surgery [44]. The term has recently been adopted in obstetrics as ERACS [44]. The components of ERACS include preoperative patient education to improve patient involvement in managing their expectations and compliance with the ERACS protocol, reduced hours of fasting, multimodal analgesia, limited use of opioids, and scheduled nonopioid analgesics and therapies to reduce pain [41]. However, in Indonesia, ERACS was promoted as an “advanced” CS technique claimed to expedite women’s recovery after CS within 24 hours and was accessible for women who wanted to have a painless birth. We observed that inadequate information was shared regarding ERACS, where only the benefits were mentioned without details on potential risks and under which indications women were able or suggested to receive ERACS.

The advertisements included in this study have the potential to influence beliefs that CS is superior to vaginal birth. For example, our study observed that most products advertised on Instagram aimed to convey that faster CS is possible, and these products were “natural” medicine for CS. Feng shui practices were actively promoted, whereby selecting an auspicious day and time for birth was recommended for the baby’s benefit—a practice favoring planned CS [9,45,46]. One woman who had elective CS at a private facility even shared a story that there were 24 other women in the queue waiting to have CS on that day as it was a “pretty” date (referring to September 19, 2019). Moreover, unbalanced promotion of ERACS may also give the impression to women that painless birth with ERACS is a better alternative compared to vaginal birth. Finally, the uncertainties and lack of control due to the COVID-19 pandemic may have exacerbated and influenced women’s preferences for CS compared to vaginal birth [14]. There is a need to counteract these unbalanced advertisements with more neutral and credible information about birth options, enabling women to make informed choices and participate actively in their care.

Our analysis shows that women may be exposed to misinformation about CS on social media and may have limited capacity to distinguish between trustworthy and untrustworthy sources of health information. Misconceptions can emerge from unregulated sources of information, with the potential for substantial impacts on behavior and decision-making. While social media is not the only source of misinformation, it has a massive reach and capacity to influence health behaviors. For example, an analysis of diet and exercise posts on Instagram showed that influencers actively promoted diet supplements and sportswear fashion as key to attain ideal body shapes and improve happiness, which has the potential to negatively impact young people’s mental and physical health and development [47]. In fact, a recent study found that more time on Instagram led to increased body dissatisfaction, frequent appearance comparisons, and lower self-esteem [48]. Similarly, the WHO’s recent report on exploitative marketing by the formula milk industry showed that women were persistently targeted using personalized approaches—including via social media influencers—to dissuade exclusive breastfeeding [49]. This is despite the International Code of Marketing of Breastmilk Substitutes banning the advertisement of breast milk substitutes to the public [49]. These examples show that, similarly to CS, health misinformation on social media is common. According to San Cornelio [15], information on Instagram typically “tend to mimic hegemonic discourses rather than create resistant alternatives.” Therefore, more work is needed to combat health misinformation via evidence-based health communications and exploration of regulations to limit the spread of misinformation.

Furthermore, advertising is part of the consumerism societal model, where “selling more to make more profit” is nonnegotiable despite most of what is sold does not have evidence supporting it. Alarmingly, the power of propaganda in advertisements with regard to birth is based on the notion of “maternal sacrifice” [50]—the fact that women naturally put their baby’s health first. Maternal sacrifice has been long used to justify certain ideologies or practices, where women were given a choice yet the idea of maternal sacrifice was deployed to influence women in making the “right choice,” invoking women’s guilt when they did not pursue it [50]. This phenomenon was also observed in a study of maternity discourse on Spanish Instagram, where intensive mothering was promoted, encouraging women to make choices deemed “best for the baby” with limited consideration for the woman herself [15]. Aligned with the consumerism societal model, we observed that no posts were made by public health facilities or providers working in the public sector in relation to CS, which is similar to a previous study on Spanish Instagram that demonstrates the absence of information surrounding maternity from governmental organizations [15]. This could be due to the need to generate profit in the private but not the public sector [51]. Health information dissemination from government organizations may be essential to counteract informational biases favoring CS over vaginal birth.

It is important to note that we identified many posts with CS hashtags that were unrelated to CS. Although these posts were not related to CS, CS hashtags were used possibly to gain a wider audience, especially women in their reproductive age regardless of pregnancy status—a common phenomenon called “hashtag hijacking” [52]. This suggests that CS-related hashtags may be a popular hashtag searched among women, which may prompt sellers and advertisers to use CS hashtags in their posts as an effective broadcast strategy. We also observed that CS posts used partnerships with influencers to increase their posts’ credibility, showing that *who says it* is more important than *what it says* in changing behavior. Thus, engaging influencers when delivering social media–based interventions may be imperative to promote visibility and change.

Finally, we found that some women felt devalued for undergoing CS and were not considered “complete mothers” because they did not experience labor. The existence of stigma attributed to CS and increasing CS seems paradoxical; however, this was similarly observed in Mexico, where CS was reported to be >45% [20]. In Mexican media Facebook pages, users commonly used derogatory terms to describe women who had CS and considered women who underwent vaginal birth as braver and stronger [20]. In Indonesia, sociocultural and religious beliefs may influence societal norms to glorify natural birth [53]. These beliefs and norms, coupled with the recent rise of CS in Indonesia [7], may result in stigmatization of women who undergo CS. Through this stigmatization, women reported feeling inadequate, disconnected, and powerless during childbirth when they failed to meet societal expectations of a drug-free vaginal birth [16]. However, we identified increasing posts that aimed to debunk the stigma placed on women who underwent CS, indicating that there seems to have been a shift on how society perceives CS in recent years. However, messages need to be evidence informed and include the risks and benefits of different modes of birth to ensure that CS is not unintentionally promoted as superior to vaginal birth. Furthermore, any health promotion should carefully consider terms or phrases that may contribute to further stigmatization of women based on their mode of birth.

### Strengths and Limitations

To our knowledge, this is the first study exploring the portrayal of CS in Indonesia through Instagram. It used modern machine learning approaches that enabled us to analyze large quantities of data and adopted a mixed methods approach that enabled us to explore surface- and in-depth–level data. However, our study has some limitations. Jakarta was the predominant location of the posts, which is the region with the highest gross domestic product in the country where most business activities happen and economic agglomeration occurs [54] and makes it more difficult to determine whether the portrayal of and discourse on CS are similar in other parts of Indonesia. We were also unable to conduct facial analysis on images due to ethical considerations (anonymity, privacy, and credibility on emotion analysis) even though the most frequent objects appearing in photos were people. However, we compensated via a thorough qualitative analysis to explore themes in depth.

### Implications for Future Research and Practice

More efforts are needed to regulate service advertisements by health care providers and facilities in the country and mobilize the regulations enforcement to ensure that women receive credible and evidence-based information that is beneficial to them. There is also a need to promote and disseminate clear, transparent, factual, and unbiased information regarding mode of birth on social media, including more research on the most efficient way to disseminate this information and what the impact is. More evidence is also needed to understand women’s perceptions and preferences regarding different modes of birth in Indonesia. Additional studies to investigate how social media influences mode of birth uptake will be useful to identify areas of improvement on social media communication regarding birth.

### Conclusions

Our study revealed how CS was portrayed in Indonesia through Instagram. Most Instagram posts about CS aimed to advertise products or services, which may influence women to perceive that CS is a better alternative than vaginal birth and that inconveniences can be easily addressed. This study highlights the need for advertisement regulation and its enforcement regarding birth-related medical services and more health promotion aiming to provide accurate, balanced, and appropriate information for women regarding mode of birth.

## Appendix

Multimedia Appendix 1 This includes hashtags used to identify caesarean section posts, coding domains for data extraction, and a flowchart of data retrieval, cleaning, and sampling. It also visualizes common objects, tags, color combinations, bigrams, and hashtags, provides a glossary of Bahasa Indonesia terms with English translations, and categorizes posts tagged with caesarean section hashtags that were not relevant to the topic.

## Abbreviations

API: application programming interface
CS: cesarean section
ERACS: enhanced recovery after cesarean surgery
VBAC: vaginal birth after cesarean section
WHO: World Health Organization

## Data Availability

The data sets generated during and analyzed during this study are available from the corresponding author on reasonable request.
