# Supplementary material for: The Portrayal of Cesarean Section on Instagram: Mixed Methods Social Media Analysis
Source: JMIR Form Res. 2024 Sep 6;8:e46531. doi: 10.2196/46531 (PMC11415719; doi:10.2196/46531)
Supplement: Multimedia Appendix 1 [file formative_v8i1e46531_app1.docx]

# Multimedia Appendix 1

## Table S1. List of #hashtags used to search for caesarean section posts

| Category | Bahasa Indonesia words | English translation |
| --- | --- | --- |
| Caesarean birth | #kelahirancaesar | Caesarean birth (kelahiran comes from the word of “lahir”, which means birth; caesar means caesarean) |
|  | #lahirancaesar  #lahirancesar  #lahiransc  #lahiransecar  #lahiransesar | Caesarean birth (lahiran comes from the word of “lahir”, which means birth; sc is the short version of caesarean, while caesar, cesar, secar, and sesar are different spellings of caesarean) |
|  | #melahirkancaesar  #melahirkancesar  #melahirkansc  #melahirkansecar  #melahirkansesar | Caesarean birth (melahirkan comes from the word of “lahir”, which means birth; sc is the short version of caesarean, while caesar, cesar, secar, and sesar are different spellings of caesarean) |
|  | #melahirkansecaraoperasi | Giving birth through surgery [caesarean birth] (melahirkan comes from the word “lahir”, which means birth; secara means through; operasi means surgery) |
| Caesarean surgery | #operasicaesar  #operasisesar | Caesarean surgery (caesar and sesar are different spellings of caesarean; operasi means surgery) |
|  | #operasicaesaribuhamil | Caesaren surgery of pregnant women (caesar means caesarean section; operasi means surgery; ibu hamil means pregnant women) |

## **Table S2. List of coding domains used for extraction.**

| Coding domains used for extracting Instagram posts |
| --- |
| Metadata and post-type  We extracted location of the post owners (if available), date of the post (reclassified as pre- or during COVID-19 pandemic), and type of the post (birth story, health message, or advertisement).  Advertisements  We categorised posts that were advertisements, operationally defined as posts aimed to promote services or products relevant to CS. This included type of products offered and type of institutions doing the advertising (commercial company, private or public health facility, individual health workers or sellers, and individual non seller).  Health messages  We classified posts aimed at sharing or giving health information (i.e., tips, indications, overview) as health messages. The health messages related to CS were coded based on the content of health messages and type of poster. Where health messages were posted by healthcare providers or a health facility account (as claimed by the accounts), we categorized these posts as “health messages from trusted sources”. If accounts other than healthcare providers or health facility posted content, we classified these as “health messages from non-trusted sources”.  Birth stories  We coded women’s birth stories, including types of health facilities where women gave birth and their mode of birth. We inductively analysed the story told by women on the image post caption.  Descriptive content of image, caption, and tags  We extracted type of image posted (people or objects), identified demographic characteristics of the people posted (gender, age, profession), post captions, #hashtags, and emotions portrayed based on the image and text. This data was then summarised and thematically analysed to explore the identified themes discussed in the text, including emotions and overall meaning of the caption together with the image.  Positionality about CS  We defined positionality about CS as whether the post explicitly or implicitly promoted or encouraged CS. The specific definitions for each category were:   - Explicitly encouraging: poster strongly promoted or encouraged CS irrespective of medical facts or conditions - Implicitly encouraging: poster talked about CS benefits or a having a positive experience with CS - Neutral: poster did not preference either CS or vaginal birth - Implicitly discouraging: poster discussed CS risks or having a negative experience with CS - Explicitly discouraging: poster discouraged CS use |

## **Figure S1. Flowchart depicting data retrieval, cleaning, and sampling.**

**
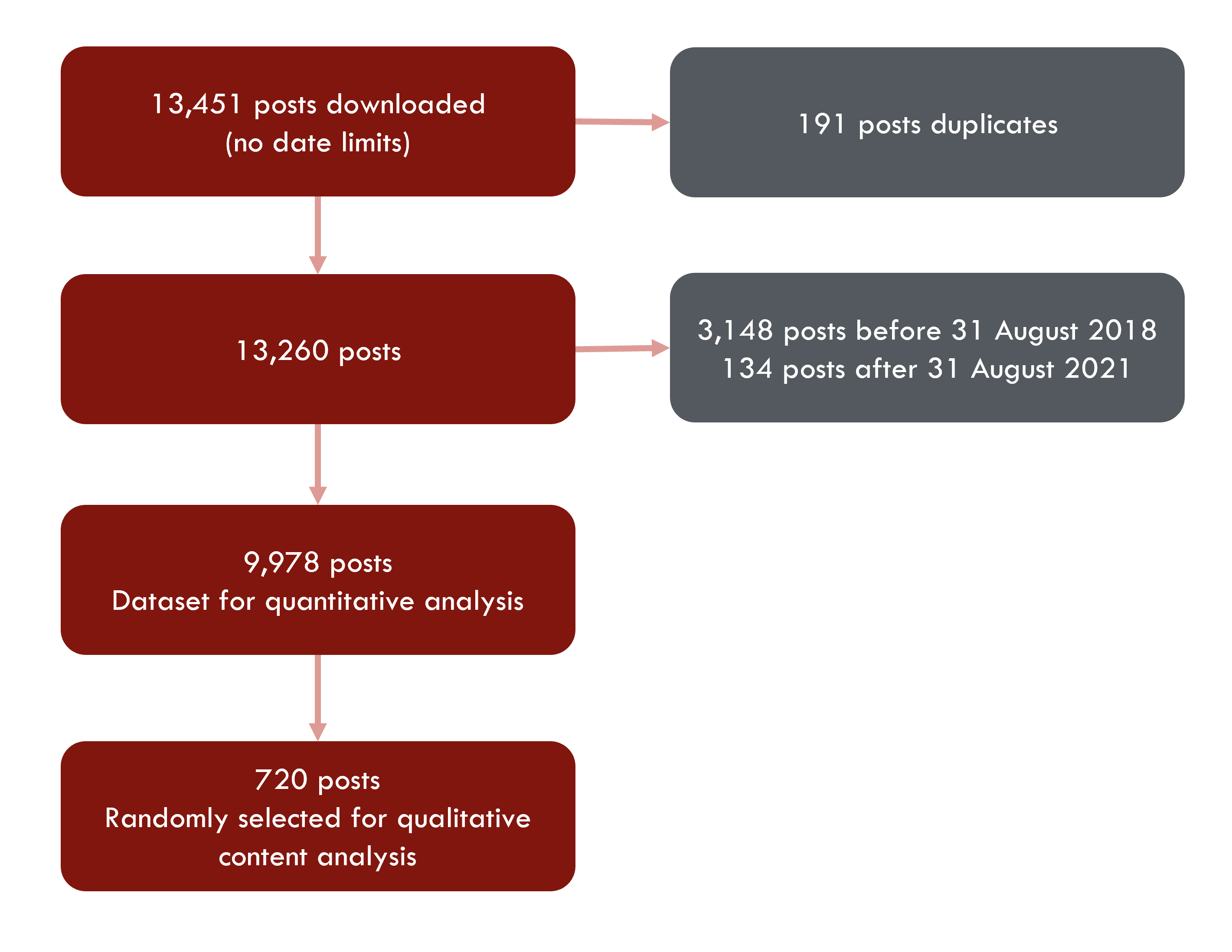
**

## **Figure S2.** 25 most frequent objects, tags, and colours combinations used in caesarean section posts

## **
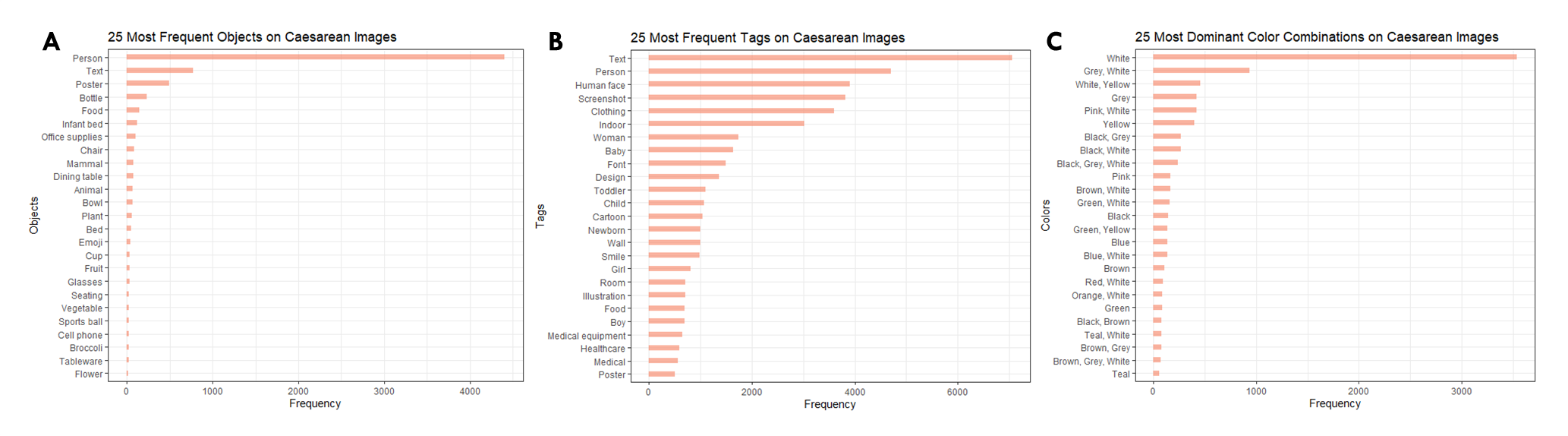
**

## **Table S3. Glossary of example words in Bahasa Indonesia with English translations**

| Category | Bahasa Indonesia words | English translation |
| --- | --- | --- |
| Words relating to advertised products for faster recovery | Channamix | Brand name of supplements for faster caesarean recovery |
|  | Kapsul kutuk | Herbal medicine brand for caesarean wound recovery |
|  | Lebih cepat | Faster [recovery] |
|  | Obat herbal | Herbal medicine |
|  | Pengobatan | Treatment |
|  | Penyembuhan luka | Wound recovery |
|  | Proses penyembuhan | Recovery process |
|  | Royalmix | Brand name of supplements for faster caesarean |
| Most frequent #hashtags appearing on caesarean images | #InginHamilInstan | “want to get pregnant instantly” |
|  | #KapsulKutukPremium | Herbal medicine to expedite caesarean recovery |
|  | #LukaOperasi | [caesarean] surgery wound |
|  | #MelahirkanCaesar | Caesarean birth |
|  | #MelahirkanNyaman | Comfortable labour |
|  | #ObatCaesarAlami | Natural medicine for caesarean |
|  | #OperasiSesar | Caesarean surgery |
|  | #PersalinanLancar | Smooth labour |
|  | #5xLukaCaesarCepatKering | Caesarean wound heals 5 times faster |
| ****#Hashtags related to ERACS**** | #ERACS | ERACS |
|  | #OperasiCaesarSecaraERACS | Caesarean with ERACS |
|  | #OperasiERACS | Caesarean with ERACS |
|  | #OperasiSesarSecaraERACS | Caesarean with ERACS |
|  | #OperasiTanpaNyeri | Surgery without pain |
|  | #OperasiTanpaSakit | Surgery without pain |
|  | #SCSecaraERACS | Caesarean with ERACS |
|  | #SesarSecaraERACS | Caesarean with ERACS |
|  | #TeknikERACS | ERACS Technique |

## **Figure S3.** 50 most frequent bigrams on caesarean section images before and during the COVID-19 pandemic (N=9978 posts in both periods; n=4065 posts before the pandemic; n=5913 posts during the pandemic).


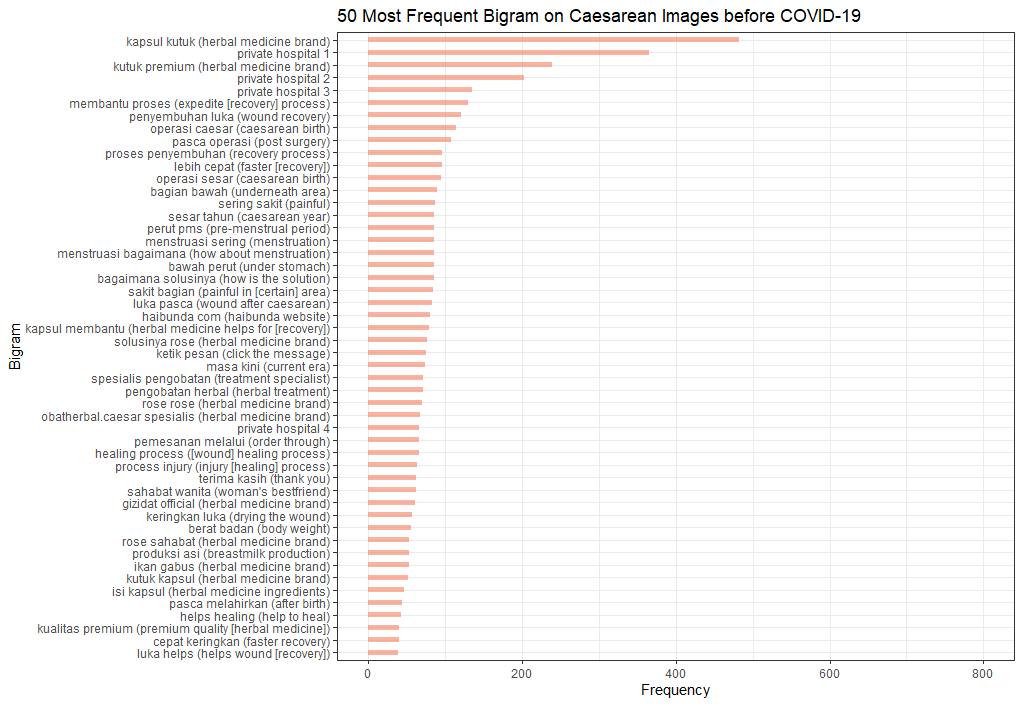


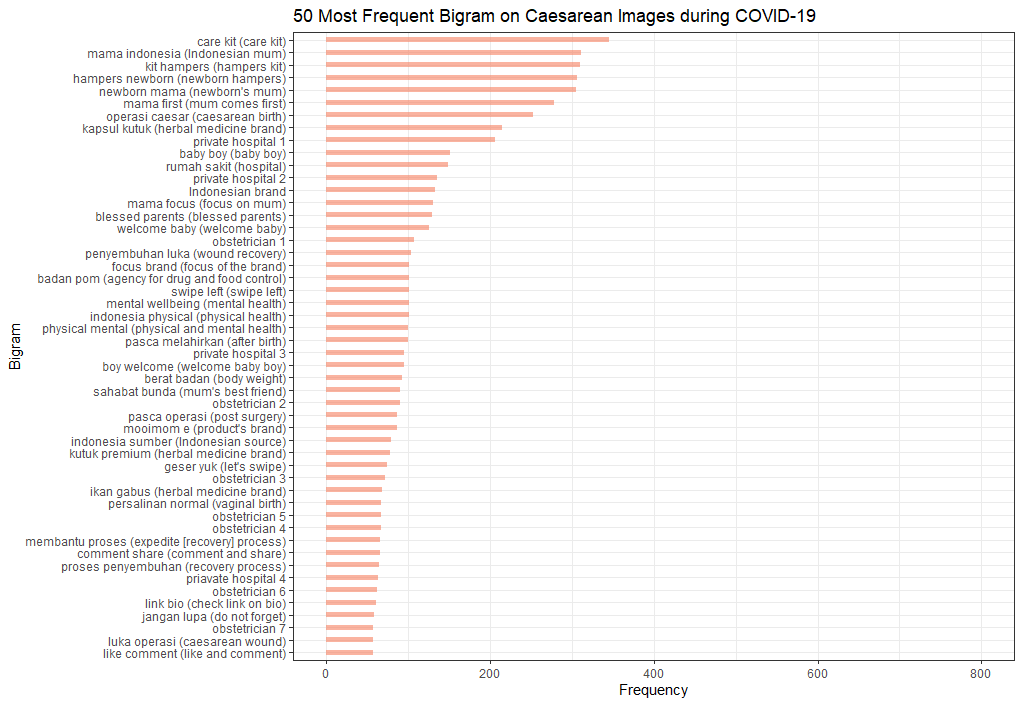


## **Figure S4.** 50 most frequent #hashtags on caesarean section posts before and during the COVID-19 pandemic (N=9978 posts in both periods; n=4065 posts before the pandemic; n=5913 posts during the pandemic).


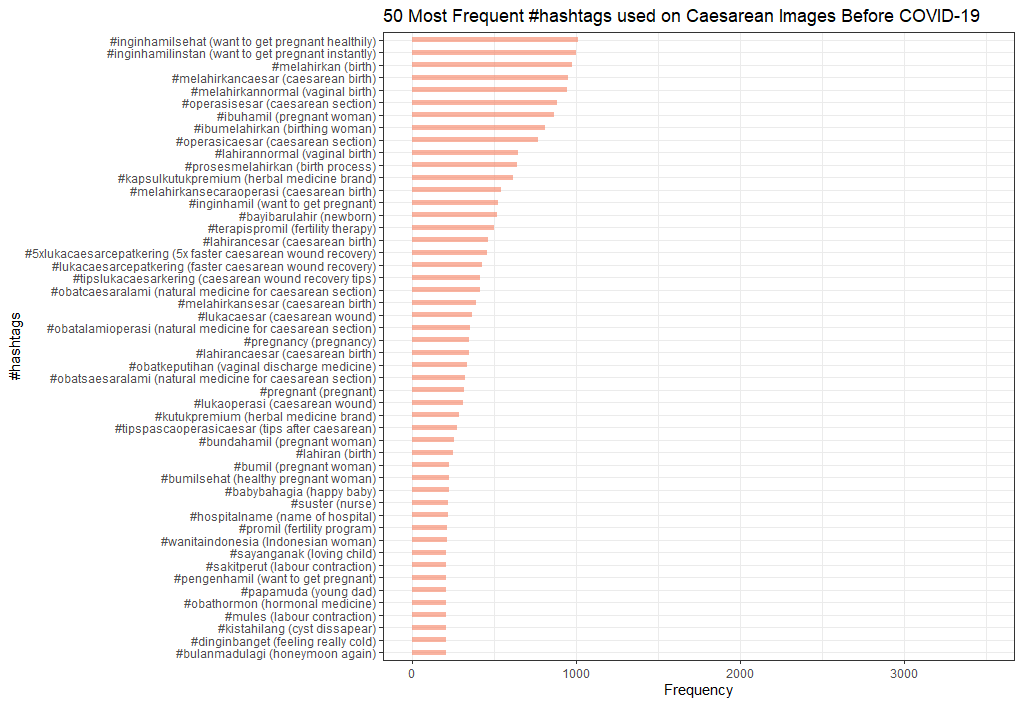


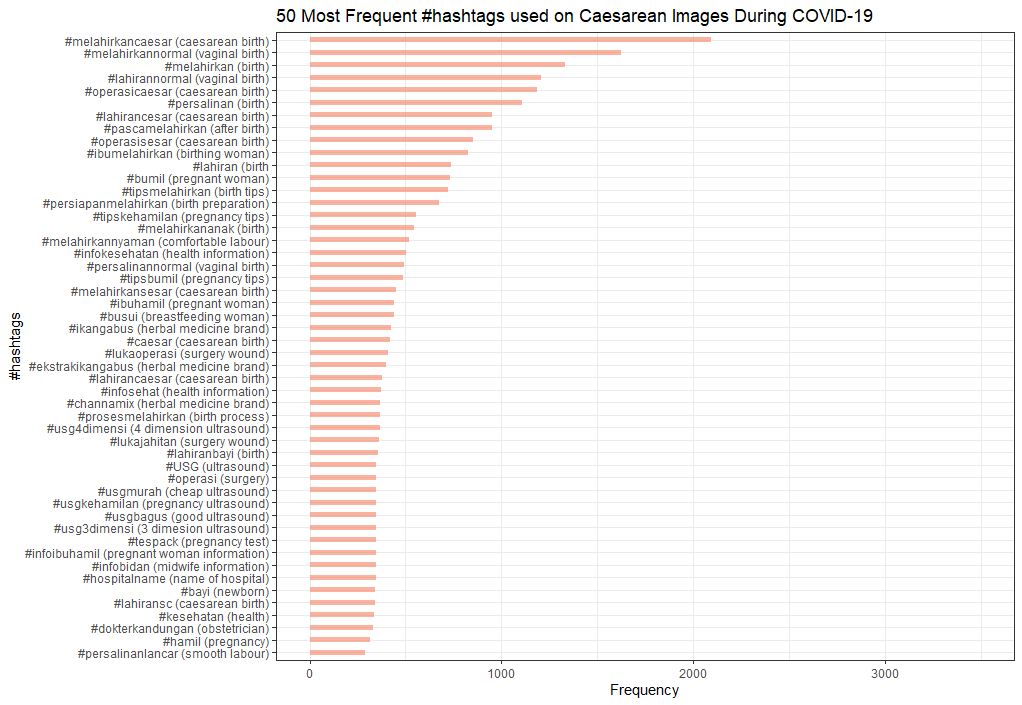


## **Table S4. Type of posts and themes emerging from posts used caesarean section #hashtags yet were not relevant to caesarean section.**

| Type of posts | Number of posts (%) | Themes of the posts |
| --- | --- | --- |
| Advertisement | 223 (56.5%) | **Promoting products to sell, which are not relevant to caesarean, which include:**   - Postpartum food catering service - Postpartum skin care product (mostly for eliminating stretchmark) - Hospital promotion (by sharing patient testimony, service promotion, facility offered, discounts, provider profile) - Medicine (herbal and non-herbal) and supplement for pregnancy, birth and postpartum - Vaginal wash product - Maternity photoshoot or birth documenting service - Infertility treatment product - Postpartum diet service and product for "returning" to normal body shapes - Breastfeeding booster product - Aqiqah (Islamic ceremony for newborns) product - Postpartum kit package and service - Gentle and hypno birth service - Baby products - Plastic surgery product advertisement - Medical instruments products (mostly advertise during COVID-19) - Other non-maternity related products (armpit cleaner, towel, phone case, multipurpose belt) |
| Trusted health messages | 50 (12.7%) | These health messages came from either clinicians, midwives, or hospitals. The posts mostly aimed to not only educate public but also advertise their names, services, and hospitals/clinics. Posts shared information (symptoms, tests, and treatments) in relating to variety of diseases - which not related to CS - as well as reminders and tips.   **The diseases covered** **include** flu, hepatitis, cirrhosis, cancer and tumor, dengue, heart disease, sexual transmitted infections (AIDS, syphilis), blood disorder (thrombocytosis), skin disorders, bones disease, diaper rash, hair loss (alopecia), hernia, rubella and measles, appendicitis, insomnia, bleeding gums, toxoplasmosis, premature birth, morning sickness, vaginal discharge, bipolar, impetigo, tuberculosis, cataracts.  **Reminders and tips include:**   - Danger of smoke from forest burn - Danger of electric cigarettes - Childbirth tips during COVID-19 pandemic - Myth about birth - Hypno birth information - Breastfeeding education - Danger of massaging pregnant women - Pregnancy tips - Jet lag tips - Reminder to exercise |
| Non-trusted health messages | 105 (26.6%) | Health messages source could not be trusted and often come to community accounts, sellers which aims to advertise their products, or individual accounts. Most messages are related to maternity including some which aim for social movement, yet there are some which are non-maternity related.  **Maternity related health messages:**   - **Pregnancy:** bonding with fetus, skin care (managing stretchmark), danger on eating sushi, pre-eclampsia symptoms, hemorrhoids during pregnancy, fetal development, mood swings, reminder that pregnancy is based on God's will - **Birth:** information on contractions and its types, hypno birth, lotus birth, national insurance coverage, urine vs rupture membrane, tax on birth, infection control, and childbirth tips (including shopping checklist, packing, documentation preparation, and birth during pandemic tips) - **Postpartum:** information about weight loss, postpartum bleeding, traditions (oukup bath, ngeboreh, jamu consumptions), postpartum care, starting sexual activity, postpartum confinement, massages, wound recovery, baby blues - **Newborn care and development:** information about breastfeeding, vitamin K injection, neonatal jaundice, how to swaddle baby, solid food and weight management, sense of day and night for baby, safety using "gurita", birth mark, myths, speech delay, shaken baby syndrome, what baby poop color means, establishing routine - **Parenting:** tips to prepare in becoming parents, dad, and returning from maternity leave - **Social movement:** encouragement to improve partner's involvement during childbirth and postpartum, being kind to other moms, and strength of human body in accommodating changes   **Non-maternity related messages:**   - Benefits of grape, green tea, apple, bitter bean, dragon fruit, cabbage, and dates to range of health conditions |
| Birth story | 5 (1.3%) | Classified as birth story due to images which depict newborns or birth process, however, no sufficient detail can be extracted to understand the mode of birth. |
| Non-birth story | 12 (3.0%) | Individual sharing their non-birth stories, which include:   - Sharing family portraits - Experience of having kids and breastfeeding - Sharing about their babies, through quote, poem and figures - Meme about pregnancy, including comparing about first vs second pregnancy |
